# The complete mitochondrial genome of a Green macroalgae species: *Ulva meridionalis* (Ulvales: Ulvaceae)

**DOI:** 10.1080/23802359.2020.1715856

**Published:** 2020-01-21

**Authors:** Xinyu Kang, Jinlin Liu, Xiaoqian Yang, Jianjun Cui, Lijuan Zhao, Qinlin Wen, Meilin Fu, Jianheng Zhang, Peimin He

**Affiliations:** a College of Marine Ecology and Environment, Shanghai Ocean University, Shanghai, China;; b North China Sea Environmental Monitoring Center, State Oceanic Administration, Qingdao, China;; c College of Fisheries, Guangdong Ocean University, Zhanjiang, China;; d Department of Marine Sciences, Co-Innovation Center of Jiangsu Marine Bio-industry Technology, Lianyungang, China

**Keywords:** *Ulva meridionalis*, Green algae, mitochondrial genome, phylogenetic analysis

## Abstract

*Ulva meridionalis,* a green macroalgae, is one of the causal species for green tides in Japan and spread into the coast of China. During this research, we sequenced the complete mitochondrial genome of *U. meridionalis*. The mitogenome is 62,887 bp in length, including 28 encoding genes and 29 tRNA genes. Compared with the *Ulva* species from mitogenome, the gene order and organization of this mitogenome are similar to most of other determined *Ulva* mitogenomes, with the nucleotide base composition of A 33.6%, T 32.2%, C 16.2%, and G 18.0%. Phylogenetic analysis shows *U. meridionalis* is closely related to *Ulva flexuosa*.

Species causing large-scale green tide disasters worldwide are mainly belong to the genus of *Ulva* (Blomster et al. 2002; Smetacek and Zingone 2013; Zhang et al. 2019). The classification of *Ulva* is complex and has been a research hotspot (Ayden et al. 2003). *Ulva meridionalis* was a large marine tropical macroalgae that mainly distributed in southern Japan (Shimada et al. 2008; Horimoto et al. 2011). This species was never observed in the Yellow Sea of China before. However, in 2019, we discovered that this species widely distributed in the crab aquaculture ponds near the estuary of Dagu river, Jiaozhou-bay area of Qingdao, China (36°12′26.316″N, 120°06′37.756″E), and the specimen was stored in the herbarium of Shanghai Ocean University Museum (SHOU2019QD082001). This species had a strong ecological adaptability, the maximum growth rate can reach up to 0.796 d^−1^ under the conditions that nitrogen and organic phosphorus concentrations are 4.02 mg/L and 0.125 mg/L (Yang 2018), which have a high risk to become a new green tide in China, so it is of great significance to sequence the mitochondrial genome to understand its evolutionary relationship.

After sample collection, the sample was cultured in the laboratory with VSE medium at 20 degrees Celsius under a light intensity of 100 μmolm^−2^s^−1^ (Jiang et al. 2019). The complete mitochondrial genome of *U. meridionalis* is 62,887 bp in length (Genbank accession number: MN861072). The overall base composition of mitochondrial genome is A (33.6%), T (32.2%), C (16.2%), G (18.0%), similar to most of the *Ulva* macroalgae in mitogenome, and the percentage of A + T (65.8%) is higher than C + G (34.2%). It contains 28 encoding genes and 29 tRNA genes. A Maximum-likelihood (ML) phylogenetic tree with 11 species complete mitochondrial genome of *Ulva* species and one outgroup (*Pseudendoclonium akinetum*) was constructed in MEGA 7 software (Figure 1) (Kumar et al. 2016), which shows *U. meridionalis* is closely related to *Ulva flexuosa*.

**Figure 1.**
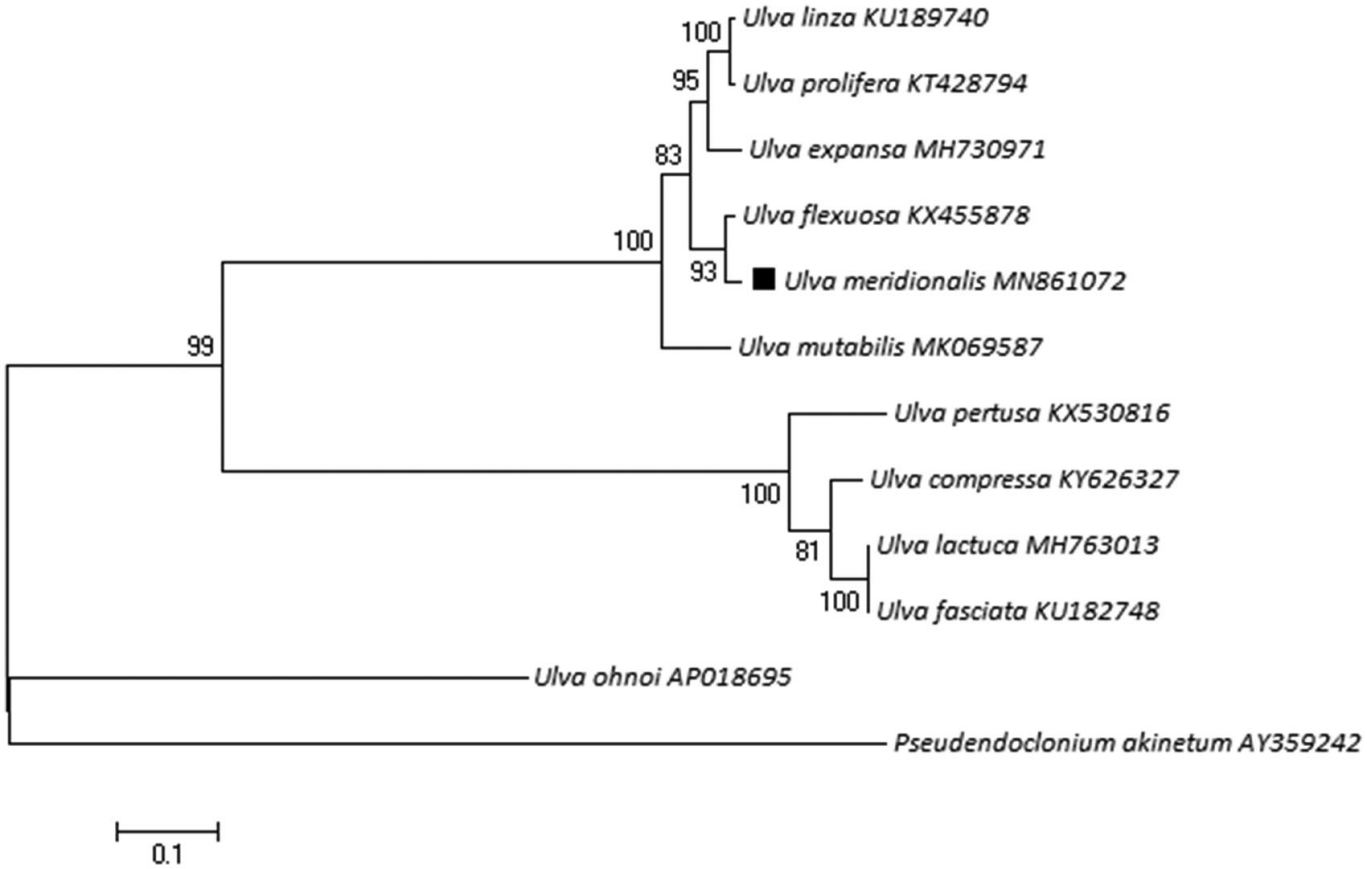
The phylogenetic tree relationship of 12 species in Phylum Chlorophyta based on the data of encoding genes. Genbank accession numbers: *Ulva meridionalis*: MN861072; *Ulva compressa*: KY626327; *Ulva expansa*: MH730971; *Ulva fasciata*: KU182748; *Ulva flexuosa*: KX455878; *Ulva lactuca*: MH763013; *Ulva linza*: KU189740; *Ulva mutabilis*: MK069587; *Ulva ohnoi*: AP018695; *Ulva pertusa*: KX530816; *Ulva prolifera*: KT428794; *Pseudendoclonium akinetum*: AY359242.
